# Vitamins and Melanoma

**DOI:** 10.3390/cancers7030841

**Published:** 2015-07-24

**Authors:** Irene Russo, Francesca Caroppo, Mauro Alaibac

**Affiliations:** Unit of Dermatology, University of Padua, Via Battisti 206, 35128 Padua, Italy; E-Mails: irenerusso88@yahoo.it (I.R.); francesca.caroppo@studenti.unipd.it (F.C.)

**Keywords:** vitamins, melanoma, cancer

## Abstract

A tremendous amount of information was published over the past decades in relation to the role of vitamins in various neoplastic diseases. In particular, several studies showed an inverse relationship between selected vitamins intake and cancer risk. In this review we will focus on the role played by vitamins in melanoma with particular regard to vitamin A, D, K, E and C. Given that vitamin supplementation is easy, convenient, and readily accepted by patients, in the future the use of vitamins in chemoprevention and therapy of melanoma could be encouraged if supported by pre-clinical and clinical evidence.

## 1. Introduction

Cutaneous melanoma is a life threatening skin tumour. In the last decades, its incidence has increased faster than for any other solid tumour [1]. Melanoma is one of the most frequent cancers in Caucasian populations [2] with a global incidence of 160,000 new cases per year and 48,000 deaths [1]. In Australia yearly incidence of melanoma is 56 cases per 100,000 per year for men and 41 cases per 100,000 per year for women [1], and among US white people is about 19 cases per 100,000 per year for men and 14 cases per 100,000 per year for women [3]. In Europe, some of the highest incidence rates are found in Austria (21.5 cases per 100,000 for men and 17.5 cases per 100,000 for women) [1]. In parallel with the increased incidence rate, an increase of melanoma related-mortality has also been described [2].

Melanoma risk depends on genetic and environmental factors [1]. Solar ultraviolet (UV) exposure, especially when intermittent and associated with sunburns, is the most important risk factor for the development of cutaneous malignant melanoma [4]. Host factors are also important, notably number of naevi [5], pigmentary characteristics (such as hair, eye and skin colour and Fitzpatrick classification of skin’s sensitivity to sunburns and ability to tan), family history of melanoma and actinic damage indicators [6].

Vitamins are organic compounds and essential nutrients for the body’s growth, development, differentiation and protection. Most vitamins cannot be synthesized in sufficient quantities by human body, thus they must be obtained from the diet. Vitamins can be divided into two groups: Water-soluble vitamins (B-complex vitamins and C vitamins) and fat-soluble vitamins (A, D, E and K). Although water-soluble vitamins need regular replacement in the body, fat-soluble vitamins are stored in the liver and fatty tissues and are eliminated much more slowly than water-soluble vitamins. Anticancer properties of lipophilic vitamins have been investigated in sevral studies [7,8,9,10,11,12,13,14,15]. In particular it has been investigated the efficacy of tretinoin against clinical promyelocytic leukemia [7], the antiproliferative effect of vitamin D in breast cancer and colorectal cancer [8,9,10], the apoptosis-inducing and chemosensitivity-enhancing effects of vitamin E succinate in bladder cancer cells [11] and the antitumor effects of vitamin K against hepatocellular carcinoma [12]. In this review, we will discuss the relationship between melanoma and vitamins, especially A, D, E, K and C.

## 2. Vitamin A

Vitamin A (retinol) is a fat-soluble organic substance that cannot be synthesized by humans. Retinol is usually found in eggs, milk, liver and plant-based pro-vitamin A carotenoids that are rich in retinoic acid esters [16]. Vitamin A is necessary for normal physiologic function, and thus, it has been classified as an essential nutrient. Vitamin A belongs to a class of compounds called retinoids that includes retinaldehyde and retinoic acid, as well as a large number of synthetic compounds. All of the retinoids are composed of three units: a hydrophobic region, a linker region, and a polar region (usually a carboxylic acid) [17].

Retinoic acid has been demonstrated to inhibit several biological functions, including tumor growth, angiogenesis and metastasis [17]. Retinoids seem to be effective in inhibiting proliferation and inducing apoptosis and differentiation [18]. It is believed that the inhibitory effects of retinoic acid are obtained through the activation of retinoic acid receptor (RAR) or retinoic X receptor (RXR). After ligand binding, RAR and RXR are transferred into cell nuclei and bind to the retinoic acid response elements (RARE), activating down-stream gene expression [19]. The activation of classical retinoid pathway is responsible for cellular differentiation, arrest, and eventually, apoptosis [20]. Retinoic acid may regulate the down-stream gene expression not only by classic pathway, but also through the modulation of several transcription factors, such as NF-kB, IFN-γ, TGF-β, MAPK [18,21]. Recently it has been shown that the retinoic acid may also regulate stem cell differentiation [22]. 

With regard to the relationship between retinoic acid and melanoma, it has been demonstrated that retinoic acid are able to inhibit growth of murine and human melanoma cell lines [23,24,25,26]. These effects are probably mediated by the activation of cyclic AMP-dependent protein kinase and sialyltransferase [21,27]. Moreover, vitamin A has been shown to inhibit human melanoma tumor cell invasion [28] by regulation of EGFR expression which is fundamental for both growth and invasion [29]. Furthermore, intercellular adhesion molecule gene I (ICAM-1) is transcriptionally regulated by retinoic acid in melanoma cells [30]. Sengupta *et al.* found that retinoic acid may also inhibit highly metastatic B16F10 melanoma cells by down-regulation of integrin receptors for laminin and other extracellular matrix proteins [31]. 

Vitamin A has also antioxidant properties [32] and is responsible for the reduction of the risk of ultraviolet (UV) light-induced skin tumors in mice [33]. In particular, it has been clearly demonstrated that UV may alter the metabolism of retinoid acid by reducing cellular retinol in human keratinocytes and melanocytes [34]. Therefore, systematic administration of vitamin A has been proposed as a melanoma chemoprevention approach. Although several epidemiologic studies concerning the association between vitamin A intake and melanoma risk have been made, results are still controversial [35,36,37,38]. A recent meta-analysis of randomized controlled trials could not find a correlation between beta-carotene (a precursor of vitamin A) and melanoma risk [39]. In a recent prospective study, Asgari *et al.* found an inverse association between supplemental intake of retinol and melanoma risk [16]. The risk reduction was statistically significant only for high-dose retinol users (>1200 μg/day) [16]. In particular, this study suggested a relationship between retinol intake and the anatomic location of melanoma, in particular for cutaneous melanoma observed in sun-exposed sites [16]. Finally, in 2014 a meta-analysis demonstrated no association between β-carotene intake and melanoma risk, consistent with the results of a previous meta-analysis of randomized controlled trials [39,40] and indicated that only the intake of retinol, but not of total vitamin A or β-carotene, was significantly associated with a reduction of the risk of melanoma. 

In conclusion, vitamin A has been demonstrated to inhibit murine and human melanoma cell lines growth *in vitro* [23,24,25,26]. Moreover, vitamin A has been shown to inhibit human melanoma tumor cell invasion [28] and intercellular adhesion [30]. Finally, several studies have investigated the association between vitamin A intake and melanoma risk has, but the findings results have been controversial.

## 3. Vitamin D

Vitamin D is a fat-soluble organic micronutrient that human organism cannot synthetize. Vitamin D levels in humans depend on ultraviolet B (UVB) exposure and, to a minor extent, on the diet and supplements [41,42]. Vitamin D obtained from sunlight exposure and diet needs two idrossilations to be converted into the biologically active form (vitamin D3). The natural form of vitamin D, which is introduced with the diet, is first metabolized to 25-hydroxyvitamin D [] in the liver, and then to its active form, 1,25-dihydroxycholecalciferol [1,] or vitamin D3 in the kidneys. 1,25(OH)D, also known as calcitriol, is essential for calcium homeostasis; it also inhibits cell proliferation and induces differentiation of multiple cell types in various organs [43,44]. Furthermore, vitamin D has been demonstrated to have a high therapeutic and prophylactic potential in osteoporosis, psoriasis and autoimmune disease [45,46].

To express its biological activity, vitamin D3 needs to bind vitamin D receptor (VDR). VDR heterodimerizes with the retinoid X receptor (RXR) and translocates to the nucleus regulating the expression of many genes involved in the mechanism of cell differentiation, progression and apoptosis [47,48,49,50].

Solar exposure is essential for the production of vitamin D by the skin. In particular, UVB (280–320 nm) induce the photolysis of the B ring in 7-dehydrocholesterol (7-DHC). The resulting pre-vitamin D3 isomerizes by non-enzymatic reaction producing vitamin D3 (cholecalciferol, (3β,5Z,7E)-9,10-secocholesta-5,7,10(19)-trien-3-ol). Vitamin D3 formation is influenced by both skin phenotype and UVB dose [51,52]. On the other hand, UV radiation is also the principal risk factor involved in the development of skin cancer, including melanoma. Melanin pigment synthesis in the skin represents a natural protective mechanism against UV-induced damage and carcinogenesis [53], but also limits synthesis of vitamin D3 [54]. 

Among the functions of vitamin D, one of the most important is the strengthening of the immune system. This effect determines an increased innate immunity, associated with a multiple regulation of acquired immunity. An association between deficient levels of vitamin D3 and several types of malignant neoplasms, notably colon, breast and skin cancer, has already been shown [55]. Several studies have evaluated the relationship between solar light, vitamin D and skin cancer [56,57].

In recent years attention has been concentrated on the possible role of vitamin D in cancer risk reduction [58,59] and, in particular, in melanoma risk [60,61,62]. Some studies suggest that normal levels of vitamin D3 at the time of diagnosis are associated to a better prognosis in patients with melanoma [63,64]. Laboratory studies [59,65] and epidemiological investigations [66] have shown that vitamin D may reduce cancer risk through several biologic pathways. In particular, *in vitro* studies indicate that 1,25(OH)D may inhibit cell growth in human melanoma cell lines [9]. High circulating vitamin D concentration has been found to be associated with reduced melanoma progression and improved survival [67]. Furthermore, reduced vitamin D serum levels have been reported in patients with stage IV melanoma compared to those with stage I [68]. Recently, a placebo-controlled randomised phase II trial was started to evaluate the safety and toxicity of oral vitamin D in patients who have completed primary surgical treatment of melanoma [69].

In 2009 Major *et al.* have realized the first prospective study evaluating the relationship between pre-diagnostic serum vitamin D concentrations and subsequent melanoma risk [70]. Results of this study indicate no statistically significant association between serum 25(OH)D levels and melanoma [70], although there is a suggested protective association in the second quartiles compared to the lowest levels [70]. Another recent study showed no direct relationship between high or low levels of vitamin D and the occurrence and severity of melanoma [71]. Furthermore, several studies demonstrated that VDR expression in melanoma cells is more intense than in normal melanocytes [72,73]. In particular, it has been reported that polymorphisms of VDR genes are associated with the occurrence of several cancers including melanoma [74,75]. 

Results on the relationship between dietary intake of vitamin D and melanoma risk are controversial: some studies have found a reduced risk associated with high levels of vitamin D [60], others have described no relationship [76]. In 2011 Tang *et al.* found that daily supplementation with 1000 mg of calcium and 400 IU of vitamin D did not reduce the overall incidence of NMSC or melanoma in a large randomized double-blinded placebo-controlled trial [77]. In 2011 Vinceti *et al.* [78] have examined the association between vitamin D and melanoma risk through a population-based case-control study. They described an inverse association between dietary intake of vitamin D and melanoma risk, in particular among males and older subjects. A similar inverse association has also been described by a US-hospital-based case-control study [36]. In conclusion, although the association between vitamin D and melanoma risk is still the object of considerable debate in recent years, the potential effect of vitamin D on the risk of melanoma deserves careful consideration.

## 4. Vitamina E (Tocopherol)

Vitamin E refers to a group of fat-soluble antioxidant that include both tocopherols and tocotrienols [79]. It represents an important object of interest in clinical practice because its optimal intake can help to prevent the onset of free radical-related degenerative diseases notably cancer, atherosclerosis and arthritis [80]. 

In nature, there are eight compounds that have been found to have vitamin E activity: D-α-, D-β-, D-γ- and D-δ-tocopherol, and D-α-, D-β-, D-γ- and D-δ-tocotrienol. α-Tocopherol may inhibit melanin synthesis both directly inactivating tyrosinase, which is the key enzyme of melanogenesis in melanocytes, and affecting the post-translation levels of tyrosinase-related protein 1 and 2 [81]. Based on these results, Kamei *et al.* have demonstrated that other form of tocopherol (D-β-tocopherol and D-γ-tocopherol) have promising antimelanogenetic activity with less cytotoxicity at relatively high concentration [82]. 

Vitamin E succinate or α-tocopheryl succinate has been shown to be a potent inhibitor of neoplastic cells *in vitro* [83]. In particular, some *in vitro* studies have reported that vitamin E succinate may trigger apoptosis in several cell lines, including breast, prostate, intestine and liver cancer [84,85,86,87,88,89,90,91,92,93,94,95,96,97]. Furthermore, it has been demonstrated that vitamin E succinate may promote breast tumor dormancy [98]. Moreover, it has been reported that vitamin E succinate can inhibit growth and survival of melanoma cells *in vitro* [99,100,101,102,103,104]. The mechanism by which vitamin E succinate inhibits tumor growth includes modulation of protein kinase C activity, regulation of transforming growth factor beta (TGF-β) protein products, enhanced expression of TGF-β type II receptors, G_1_ cell cycle blockage, DNA synthesis arrest and the induction of apoptosis [99,100,101,102,103,104]. Recently, Malafa *et al.* have conducted the first study reporting the antimelanoma effects of vitamin E succinate *in vivo* [104]. In this study it has been demonstrated for the first time that vitamin E succinate may inhibit melanoma growth *in vivo* through the promotion of melanoma apoptosis [105]. Further *in vivo* studies of the antitumor effects of vitamin E succinate are necessary in order to confirm its potential role as a therapeutic micronutrient against melanoma [105]. 

In conclusion, vitamin E has been reported to be involved in inhibition of melanin synthesis [81]. In particular, vitamin E succinate can inhibit growth and survival of melanoma cells *in vitro* [99,100,101,102,103,104] and *in vivo* [104]. These results support a potential protective effect of vitamin E for melanoma.

## 5. Vitamin K

Vitamin K refers to a group of fat-soluble vitamins which are necessary for blood coagulation and bone metabolism [106]. Natural forms of vitamin K include vitamin K1 (VK1) or phylloquinone and vitamin K2 (VK2) or menaquinone. VK1 is synthesized by plants and is the predominant form in the diet, whereas VK2 is produced by human intestinal bacterial flora. Three synthetic types of vitamin K are known: vitamins K3 (menadione), K4 and K5. 

The antitumor effect of vitamin K has been under investigation since 1947 [107]. Several studies have demonstrated that vitamin K may inhibit cancerous cell growth *in vivo* and *in vitro* [108,109,110,111,112,113,114,115]. 

Vitamin K3, called also menadione, which is a synthetic analogue of vitamin K and acts as a provitamin, was discovered to be effective against several human cancer cell lines [109,110,116]. Moreover, menadione has been showed to have a synergistic effect when combined with conventional chemotherapeutic agents [106]. The association between menadione and 5-fluorouracil (5-FU) significantly increased the action against hepatoma cells [117], whereas the combination with 5-FU, bleomycin, cisplatin and decarbazine enhanced the action against oral epidermoid carcinoma cell culture [106]. Furthermore the combination of VK3 (menadione) and vitamin C without he concomitant use of chemotherapy and radiation has shown anticancer effects *in vitro* and *in vivo* [110]. 

Although most of the anticancer studies has focused on VK3 (menadione), there have been some investigations reporting the anticancer effects of VK1 and VK2 [106]. Phylloquinone and menaquinone seem to have anticancer activity in many cancer cell lines (colon, lung, stomach, liver, breast, nasopharynges, oral epidermoid cancer and leukemia) [118]. The anticancer effects of vitamin K may be explained through several mechanisms: oxidative effect, direct arylation of thiols resulting in the depletion of glutathione, cell cycle arrest and cell death through the inhibition of protein kinases and regulation of transcriptional factors causing the expression of c-myc and c-fos protoncogenes [106].

The antiproliferative and apoptosis-inducing activity of vitamin K derivatives has been investigated in A375 human melanoma cells, a highly metastatic human amelanotic cell line [119]. Ishibashi *et al.* observed that VK3 (menadione) and VK5 (synkamin hydrochloride) have a significant antiproliferative effect at a concentration range of less than 10 μM/L inhibiting the growth of decarbazine resistant human melanoma cells [119]. The same activity was also found in mouse eumelanotic B16F10 melanoma cells [119]. 

Menadione has been identified as a specific inhibitor of the E3 ubiquitin ligase Siah-2 which is generally up-regulated in melanoma [120]. To this regard both the Ras-Raf-MEK-ERK (MAPK) and the hypoxia signaling pathways are activated through several mechanism in melanoma and play a key role in melanoma development and progression [121,122,123,124]. The ubiquitin ligase Siah2 has been described to be implicated in the regulation of both hypoxia response and Ras/MAPK signaling, thus ubiquitin ligase Siah represents a potential target for inhibition in melanoma [125]. In conclusion, vitamin K has been reported to have antiproliferative and apoptosis-inducing activity on highly metastatic human amelanotic cell lines [119]. 

## 6. Vitamin C

Vitamin C is an important water-soluble antioxidant and enzyme cofactor present in the vast majority of plants and animals. Humans, unlike other animals, are not able to synthesize this nutrient endogenously and, consequently, must obtain vitamin C through their diet. There are two naturally occurring forms of vitamin C: the reduced form (ascorbic acid; AA) and the oxidized form (dehydroascorbic acid; DHA). Ascorbic acid is the most common form in the human body and performs many biochemical and biological functions. In recent years, a large number of investigations demonstrated that ascorbic acid at concentration of *in vivo* relevance is responsible for cytotoxicity to tumor cells both *in vitro* and *in vivo* [126,127,128] by means of oxidative damage on cancer cells [129]. In particular, melanoma cells were found to be more sensitive to acid ascorbic toxicity than other malignant cells [130]. Moreover, in B16 melanoma-bearing mice ascorbic acid supplementation was effective in preventing lung metastasis of the melanoma cells [131]. In line with this study, Cha *et al.* demonstrated that ascorbic acid supplementation inhibits growth and metastasis of B16FO melanoma in vitamin C-deficient mice [132]. To this regard, a recent investigation has demonstrated that there is a difference in plasma acid ascorbic levels between patients with metastatic melanoma and healthy controls [133]. Melanoma patients express low plasma acid ascorbic levels [133]. Furthermore it has been reported that polychemotherapy and immunotherapy may decrease plasma ascorbic acid levels in these patients [133]. The same group has also demonstrated in metastatic melanoma cells an influence of ascorbic acid on epigenetic regulatory features, in particular micro RNA expression [134]. Taken together, these studies provide a rationale in support of the use of ascorbic acid in melanoma chemoprevention and treatment.

## 7. Conclusions 

Vitamins have attracted the attention of the scientific community in recent decades, and it has become clear that they play a key role in the etiology of many diseases, including cancer. With respect to cancer, the scientific data developed so far are not conclusive but are optimistic and more studies are needed to clarify the true role of vitamins in cancer prevention and treatment. Because melanoma is a potentially life-threatening cancer, novel preventive and adjuvant therapies are needed to improve its prognosis and consequently the role of vitamins in this condition is exciting as vitamin supplementation is easy, convenient, and readily accepted by patients.
